# Supplementary material for: Bound states in the continuum in whispering gallery resonators with pointlike impurities
Source: Sci Rep. 2024 Nov 13;14:27929. doi: 10.1038/s41598-024-79094-8 (PMC11561169; doi:10.1038/s41598-024-79094-8)
Supplement: Supplementary file 1 — Supplementary Information. [file 41598_2024_79094_MOESM1_ESM.pdf]

Supplemental Material: Bound states in the continuum in whispering gallery resonators with pointlike impurities

M.A. Figueroa,[1] Vladimir Juričić,[1,2] and P. A. Orellana[1]

[1]*Departamento de Física, Universidad Técnica Federico Santa María, Casilla 110, Valparaíso, Chile*
[2]*Nordita, KTH Royal Institute of Technology and Stockholm University, Hannes Alfvéns väg 12, SE-106 91 Stockholm, Sweden*

The Supplemental Material contains: Details of the derivation of the transmission probability in Eq. (2) in the main text (Sec. S1); Analysis of the local density of states (Sec. S2); Analysis of the band structure along the ring with the periodically distributed impurities (Sec. S3).

S1. DERIVATION OF THE TRANSMISSION COEFFICIENT [EQ. (2) IN THE MAIN TEXT]

We here provide details of the derivation of the transmission, as given by Eq. (2) in the main text. The propagating waves obey the Helmholtz equation [1, 2]

$$-\frac{1}{\varepsilon}\nabla^2\psi = \omega^2\psi, \tag{S1}$$

where ε is the average dielectric constant of the medium, and ω the frequency of the wave. We remark that one can make a direct correspondence with the free particle Schrödinger equation by identifying $\nabla \to -\hbar\nabla$ and $\varepsilon \to 2m$, with $\hbar$ as the Planck's constant and m as the mass of the particle. Therefore, these results are directly applicable to the analogous quantum transport problem.

Defining the form of the wave amplitudes in the incoming and outgoing zones as

$$\begin{aligned} \Psi_I(x) =& e^{ikx} + re^{-ikx} \quad & x \in [-\infty, 0] \\ \Psi_{II}(x) =& te^{ikx} \quad & x \in [0, \infty], \end{aligned} \tag{S2}$$

and the corresponding wave amplitude in the ring as

$$\Psi_l^R(\phi) = (A_l e^{ika\phi} + B_l e^{-ika\phi}), \quad \phi \in [\phi_{l-1}, \phi_l], \tag{S3}$$

where

$$\phi_l = \frac{2\pi}{N} l - \theta, \tag{S4}$$

for $l = 1, ..., N$, $\phi_0 = 0$ and $\phi_{N+1} = 2\pi$, we derive the transmission coefficient applying the transfer matrix method.

Boundary conditions include continuity at the junction,

$$\Psi_I\big|_{x=0} = \Psi_{II}\big|_{x=0} = \Psi_1^R\big|_{\phi=0} = \Psi_{N+1}^R\big|_{\phi=2\pi}, \tag{S5}$$

and the Griffith boundary condition for the derivative of the wave amplitude at the junction [3],

$$\partial\Psi_{II}\big|_{x=0} - \partial\Psi_I\big|_{x=0} = \frac{1}{a}\left(\partial_\phi\Psi_{N+1}^R\big|_{\phi=2\pi} - \partial_\phi\Psi_1^R\big|_{\phi=0}\right), \tag{S6}$$

together with the boundary conditions at the impurities,

$$\Psi_{l+1}^R\big|_{\phi=\phi_l} = \Psi_l^R\big|_{\phi=\phi_l} \tag{S7}$$

$$\frac{1}{a}\left(\partial_\phi\Psi_{l+1}^R\big|_{\phi=\phi_l} - \partial_\phi\Psi_l^R\big|_{\phi=\phi_l}\right) = \varepsilon V_0 \Psi_l^R\big|_{\phi=\phi_l}. \tag{S8}$$

Defining M, the coupling matrix at the origin,

$$M = \begin{pmatrix} 1 - i\frac{\varepsilon V_0}{2k} & -i\frac{\varepsilon V_0}{2k} \\ i\frac{\varepsilon V_0}{2k} & 1 + i\frac{\varepsilon V_0}{2k} \end{pmatrix}, \tag{S9}$$

and the displacement matrix, D, as

$$D(\alpha) = \begin{pmatrix} e^{ika\alpha} & 0 \\ 0 & e^{-ika\alpha} \end{pmatrix}, \tag{S10}$$

we can then write the boundary conditions in the compact form,

$$D(\Delta\phi)^N D(-\theta) \begin{pmatrix} A_{N+1} \\ B_{N+1} \end{pmatrix} = (MD(\Delta\phi))^N D(-\theta) \begin{pmatrix} A_1 \\ B_1 \end{pmatrix}, \tag{S11}$$

given by the fact that the properties $D(\alpha_1+\alpha_2) = D(\alpha_1)D(\alpha_2) = D(\alpha_2)D(\alpha_1)$, $D^{-1}(\alpha) = D(-\alpha)$ and $\Delta\phi = 2\pi/N = \phi_1 + \theta$ are satisfied. Rearranging to a full cycle and defining $Q = MD(\Delta\phi)$, leads to

$$\begin{pmatrix} A_{N+1}e^{ik2\pi a} \\ B_{N+1}e^{-ik2\pi a} \end{pmatrix} = D(\theta)Q^N(\Delta\phi)D(-\theta) \begin{pmatrix} A_1 \\ B_1 \end{pmatrix}. \tag{S12}$$

We can then identify the terms in Eq. (S5) and (S6):

$$\begin{aligned} 1 + r = t = A_1 + B_1 = A_{N+1}e^{2\pi ika} + B_{N+1}e^{-2\pi ika} \\ ik\,(t - 1 + r) = ik\left(A_{N+1}e^{2\pi ika} - A_1\right) - ik\left(B_{N+1}e^{-2\pi ika} - B_1\right). \end{aligned} \tag{S13}$$

It is then straightforward to show that after diagonalizing $Q(\Delta\phi)$ and performing a straightforward algebra, the transmission probability $T = |t|^2$ is given by Eq. (2) in the main text.

The coefficients A_l and B_l can also be obtained by applying the transfer matrix method

$$D(\Delta\phi)^l D(-\theta) \begin{pmatrix} A_{l+1} \\ B_{l+1} \end{pmatrix} = (MD(\Delta\phi))^l D(-\theta) \begin{pmatrix} A_1 \\ B_1 \end{pmatrix} \tag{S14}$$

and solving for A_1 and B_1, which results in

$$\begin{aligned} A_1 &= \frac{1 - T_N(X) + iU_{N-1}(X)\left(Y + \frac{v_0}{ka}e^{-ika(\Delta\phi-2\theta)}\right)}{2\left(1 - T_N(X) + iU_{N-1}(X)\left(Y + \frac{v_0}{ka}\cos\left(ka(\Delta\phi - 2\theta)\right)\right)\right)} \\ B_1 &= -\frac{1 - T_N(X) - iU_{N-1}(X)\left(Y + \frac{v_0}{ka}e^{ika(\Delta\phi-2\theta)}\right)}{2\left(1 - T_N(X) + iU_{N-1}(X)\left(Y + \frac{v_0}{ka}\cos\left(ka(\Delta\phi - 2\theta)\right)\right)\right)}. \end{aligned} \tag{S15}$$

Here, $v_0 = \varepsilon V_0 a/2$ is the dimensionless impurity potential and the functions X and Y are defined as, respectively, the real and imaginary part of the complex function

$$Z = \left(1 - i\frac{v_0}{ka}\right)e^{ika\Delta\phi}.$$

S2. LOCAL DENSITY OF STATES

The destructive interference of the propagating states along the ring and the corresponding scattering state cancels any wave projection onto the waveguide, which eventually yields the bound states in the continuum. To capture this gradual confinement in the WGR, we calculate the local density of states (LDOS), defined as

$$\rho(k) = \int_{\text{Ring}} |\Psi_{ka}(\boldsymbol{r})|^2 d\boldsymbol{r}, \tag{S16}$$

where $\Psi_{ka}(\boldsymbol{r})$ is the wave amplitude at the ring [Eq. (S3)] with the dimensionless wavenumber ka and the corresponding frequency $\omega = k^2/\varepsilon$.

Fig. (S1) displays the LDOS versus the wavenumber (ka) for the case of a ring with a single impurity near the inversion-symmetric impurity configuration ($\theta = \Delta\phi/2$). The emerging narrow profile is interpreted as a partially confined or leaky mode (quasi-BICs). On the other hand, the dashed line shows the collapse to a delta-like profile, indicating the ring state's decoupling. Moreover, it is observed that the characteristic width decreases as the

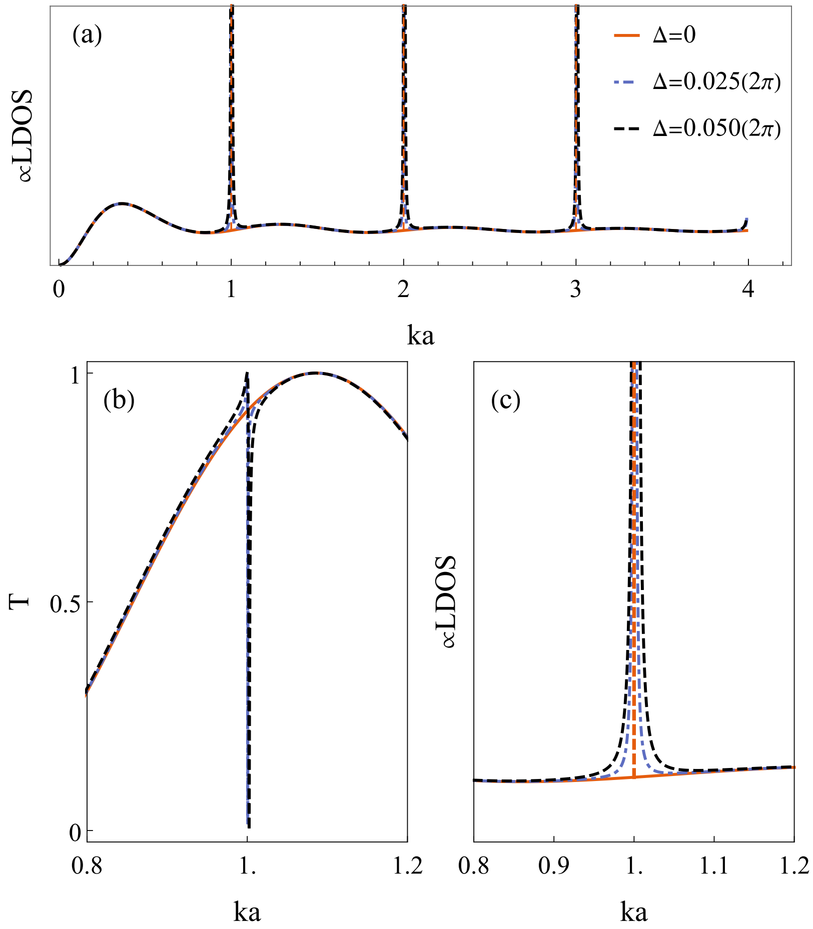

Figure S1. (a) Local density of states as a function of the dimensionless wavenumber ka for a whispering gallery resonator with a single impurity near the inversion-symmetric impurity configuration ($\theta = \Delta\phi/2 + \Delta/2$) for the dimensionless impurity potential $v_0 = 0.3$ [see Eq.(2) in the main text]. (b) Transmission amplitude and (c) LDOS for the same parameters around the lowest-energy bound state in the continuum.

coupling angle approaches the angle of the destructive interference. Figs. S1(b) and S1(c) show the conductance and LDOS, respectively, around the profile with the lowest energy for the same parameters θ and V_0 as in the Fig. S1(a). Notice that the decoupling in LDOS corresponds to the disappearance of the resonance in the conductance.

The case of three impurities, shown in Fig.4(c) in the main text, resembles the single-impurity situation. In this case, the collapse of the Fano profile accompanied by the collapse to a delta-like profile occurs for the momenta at the center of the Brillouin zone and at the coupling angle corresponding to the destructive interference.

S3. BLOCH STATES OF AN ISOLATED WGR

Here, we analyze the states of an isolated WGR in terms of the corresponding Bloch theorem, which is a consequence of the translational symmetry of the impurity configuration. We measure the distance along the WGR by the arc length x from a chosen impurity. According to Bloch's theorem, the wavefunction with the Bloch momentum q under a primitive translation by $a\Delta\phi = 2\pi a/N$, transforms as $\psi(x + a\Delta\phi) = e^{iqa\Delta\phi}\psi(x)$. In particular, in the region $0 < x < a\Delta\phi$, for such an isolated WGR, we take the wavefunction as a superposition of the forward and backward propagating plane waves with the wavenumber k and frequency $\omega = k^2/\varepsilon$,

$$\psi(x) = \left(A_+ e^{ikx} + B_- e^{-ikx}\right). \tag{S17}$$

Here, q is in the first Brillouin zone of the WGR, $q \in [-\frac{N}{2a}, \frac{N}{2a}]$, with $qa \in \mathbb{Z}$ because of the periodic boundary condition. Then, the wavefunction in the cell immediately to the left of the origin is

$$\psi(x) = e^{-iqa\Delta\phi}\psi(x + a\Delta\phi) = e^{-iqa\Delta\phi}\left(A_+ e^{ik(x+a\Delta\phi)} + B_- e^{-ik(x+a\Delta\phi)}\right). \tag{S18}$$

Continuity of the wave amplitude at $x = 0$ [Eq. (S7)], together with Eqs. (S17) and (S18), yield

$$B_- = \frac{e^{ika\Delta\phi} - e^{iqa\Delta\phi}}{e^{iqa\Delta\phi} - e^{-ika\Delta\phi}}\, A_+, \tag{S19}$$

for $\exp(\pm ika\Delta\phi) \neq \exp(iqa\Delta\phi)$, i.e. when the momenta $\pm k$ and the WGR momentum q are *not* related by a reciprocal lattice vector, or, equivalently, it does not exist an integer m such that $(k \pm q)a = mN$. Substituting this result in the condition for the jump of the derivative at the impurity [Eq. (S8)], we obtain the dispersion relation, Eq. (6), in the main text. In particular, for high-symmetry momenta, at the center of the Brillouin zone, $q = 0$, we find

$$\psi(x) = \mathcal{N}_c\left(e^{ikx} + e^{ik(a\Delta\phi - x)}\right) \tag{S20}$$

and at the edge $q = \pi/(a\Delta\phi) = N/2$,

$$\psi(x) = \mathcal{N}_e\left(e^{ikx} - e^{ik(a\Delta\phi - x)}\right) \tag{S21}$$

where $\mathcal{N}$ are the normalization constants.

When $\exp(\pm ika\Delta\phi) = \exp(iqa\Delta\phi)$, i.e., $(k \pm q)a = mN$, $m \in \mathbb{Z}$, the continuity condition is already satisfied, while discontinuity of the derivative at the impurity implies

$$B_- = -A_+. \tag{S22}$$

This is the case for the bands at the high-symmetry momenta in the Brillouin zone, where the band gap opens. At $ka = mN$, the gap opens at the center, and for $ka = N(2m - 1)/2$ at the edge of the Brillouin zone, with $m \in \mathbb{N}$. The form of the wavefunction, as implied by Eqs. (S17) and (S22), reads

$$\psi(x) = \mathcal{N}\left(e^{ikx} - e^{-ikx}\right), \tag{S23}$$

with $\mathcal{N}$ as the normalization constant. Notice that there are no solutions at the edges of the Brillouin zone for an odd number of impurities. On the other hand, from Eq. (S23), we conclude that the band gap is opened by the degeneracy splitting of symmetric and antisymmetric combinations of waves traveling in clockwise and counterclockwise directions. The position of nodes of the Bloch functions, where the BICs eventually form in the laterally coupled setup, can also be computed directly from the previous equations and the dispersion relation in Eq. (6) of the main text.

[1] El-Ganainy, R., Makris, K., Christodoulides, D. & Musslimani, Z. H. Theory of coupled optical pt-symmetric structures. *Optics letters* **32**, 2632–2634 (2007).

[2] Özdemir, Ş. K., Rotter, S., Nori, F. & Yang, L. Parity–time symmetry and exceptional points in photonics. *Nature materials* **18**, 783–798 (2019).

[3] Griffith, J. S. A free-electron theory of conjugated molecules. part 1.—polycyclic hydrocarbons. *Trans. Faraday Soc.* **49**, 345–351 (1953). URL `http://dx.doi.org/10.1039/TF9534900345`.
